# Early-pregnancy fasting blood glucose and subsequent GDM define four distinct risk groups for adverse pregnancy outcomes: a cohort study of 15,245 women

**DOI:** 10.3389/fendo.2026.1936170

**Published:** 2026-08-07

**Authors:** Jing Li, Jihong Zhang, Chun Zhang, Xuxia Liang, Yanqun Lu, Hua Wu

**Affiliations:** 1 Department of Obstetrics, Guangxi Medical Academy & Guangxi Zhuang Autonomous Region People’s Hospital, Nanning, China; 2 Department of Nutrition, Endocrinology and Metabolism, Beijing Obstetrics and Gynecology Hospital, Capital Medical University, Beijing, China; 3 Beijing Maternal and Child Health Care Hospital, Beijing, China; 4 Department of Clinical Nutrition, Capital Medical University, Beijing, China

**Keywords:** fasting blood glucose, gestational diabetes mellitus, glycemic trajectory, hypertensive disorders of pregnancy, pregnancy outcomes

## Abstract

**Objective:**

To investigate the associations between a four-group classification based on early-pregnancy FBG and subsequent GDM status and maternal and neonatal outcomes, and to characterize the FBG-HDP dose-response relationship.

**Methods:**

This retrospective cohort study included 15,245 women with singleton pregnancies. Participants were categorized into four groups based on early-pregnancy FBG (< 5.1 vs ≥ 5.1 mmol/L) and subsequent GDM status: Normoglycemia (normal FBG without GDM), GDM-Regression (elevated FBG without GDM), Late-onset GDM (normal FBG with GDM), and GDM-Maintained (elevated FBG with GDM). Multivariable logistic regression was used to estimate adjusted odds ratios (aOR) for outcomes. Restricted cubic spline regression was used to examine the dose-response relationship between FBG and HDP.

**Results:**

The incidence of HDP increased progressively across groups: Normoglycemia 4.6%, GDM-Regression 6.9%, Late-onset GDM 8.1%, and GDM-Maintained 11.0% (*P* for trend <0.001). Compared with Normoglycemia, the aOR for HDP was 1.32 (95% *CI*: 1.03–1.68) for GDM-Regression, 1.53 (95% *CI*: 1.28–1.83) for Late-onset GDM, and 1.65 (95% *CI*: 1.23–2.18) for GDM-Maintained. GDM-Regression was associated with reduced SGA risk (*aOR*: 0.69, 95% *CI*: 0.53–0.87) but elevated LGA (*aOR*: 1.45, 95% *CI*: 1.21–1.74) and macrosomia (*aOR*: 1.62, 95% *CI*: 1.19–2.17) risks. GDM-Maintained showed the highest risks for LGA (*aOR*: 1.68), macrosomia (*aOR*: 1.81), preterm birth (*aOR*: 1.78), and neonatal asphyxia (*aOR*: 2.15). Restricted cubic spline analysis revealed a nonlinear relationship between FBG and HDP (overall *P* < 0.001; *P* for nonlinearity=0.036), with a statistical inflection point at 4.52 mmol/L. Stratified analyses showed consistent associations across age, BMI, ethnicity, and parity subgroups (all *P* for interaction >0.05). Sensitivity analyses using alternative FBG cutoffs (≥5.3 and ≥5.6 mmol/L) confirmed the robustness of these findings.

**Conclusions:**

The four-group classification based on early-pregnancy FBG and subsequent GDM status may be useful for stratifying the risk of adverse pregnancy outcomes. The observed inflection point warrants further investigation. These findings support the potential value of early-pregnancy glycemic assessment in refining risk characterization for pregnant women.

## Introduction

Gestational diabetes mellitus (GDM) is the most common metabolic complication of pregnancy, with a pooled global prevalence of 14.0%, reaching as high as 20–30% in South and South-East Asia, the Middle East, and North Africa (1). Among women diagnosed with GDM, 30–70% have hyperglycemia detectable in early pregnancy, before 20 weeks of gestation (2). The association between hyperglycemia in pregnancy and adverse perinatal outcomes—including large-for-gestational-age (LGA), macrosomia, preeclampsia, and preterm birth—is well established, and randomized controlled trials have demonstrated that treatment of GDM diagnosed reduces these risks (2–4).

However, the optimal approach to early-pregnancy glycemic screening remains debated (5). Although the International Association of Diabetes and Pregnancy Study Groups (IADPSG) initially recommended classifying FPG 5.1–6.9 mmol/L before 24 weeks as GDM, this guidance was later revised this guidance due to insufficient evidence (1). While the TOBOGM trial demonstrated that early GDM treatment reduces neonatal complications, optimal diagnostic thresholds remain undefined (6). Several critical questions persist. First, what is the appropriate threshold for clinically significant early hyperglycemia? Recent evidence suggests that FPG ≥5.1 mmol/L before 20 weeks signals increased risks of hypertensive disorders of pregnancy (HDP) and fetal overgrowth, even in the absence of subsequent GDM (5, 7). Second, whether women with elevated early FPG who do not develop GDM—the GDM-Regression group—remain at increased risk has not been fully characterized (8, 9). Third, the distinct risk profiles of different glycemic trajectories—early hyperglycemia with subsequent GDM (GDM-Maintained), early hyperglycemia without GDM (GDM-Regression), and normal early FPG with late-onset GDM (Late-onset GDM)—remain poorly understood (10). While the glycemic continuum from preconception to postpartum is increasingly recognized, the clinical implications of these trajectories for maternal and neonatal outcomes require further elucidation (11, 12).

In this context, we conducted a large retrospective cohort study of 15,245 Chinese women to investigate the associations between early-pregnancy FBG and subsequent GDM status—diagnosed by 75-g OGTT at 24–28 weeks of gestation—with maternal and neonatal outcomes. Using a four-group classification based on early-pregnancy FBG and GDM status, we examined the associations of these glycemic trajectories with HDP (primary outcome) and a range of secondary maternal and neonatal outcomes. We further characterized the dose–response relationship between FBG and HDP using restricted cubic spline analysis and assessed the consistency of these associations across clinically relevant subgroups defined by age, BMI, ethnicity, and parity. By addressing these questions, we sought to provide evidence to inform risk characterization and generate hypotheses that may guide future targeted prevention strategies for women with different early-pregnancy glycemic profiles.

## Methods

### Study design and population

This retrospective cohort study was conducted at Guangxi Zhuang Autonomous Region People’s Hospital, a tertiary referral center in Nanning, China. The study protocol was approved by the Institutional Review Board of Guangxi Zhuang Autonomous Region People’s Hospital (Approval No.: KY-SY-2023-023), and informed consent was waived due to the use of anonymized clinical data.

**Figure 1:**
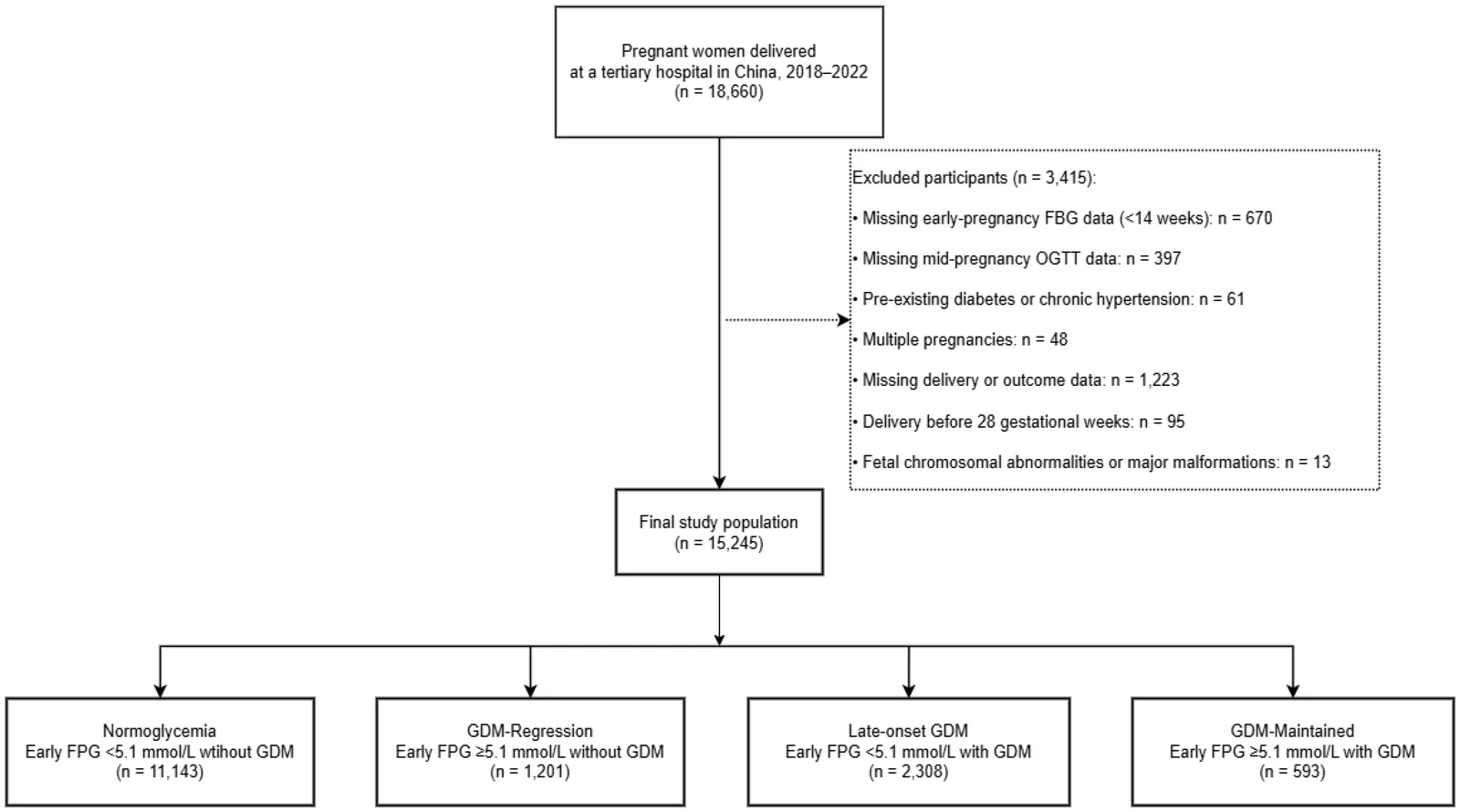
Study Population Selection Flowchart. Selection of the study population of 15,245 women with singleton pregnancies, categorized by early-pregnancy FBG and GDM status. FBG, fasting blood glucose; OGTT, oral glucose tolerance test; GDM, gestational diabetes mellitus.

We included all women with singleton pregnancies who delivered at our institution between January 2018 and December 2022. Women were eligible if they had available data on early-pregnancy fasting blood glucose (FBG) measured at < 14 weeks of gestation, completed a 75-g oral glucose tolerance test (OGTT) at 24–28 weeks of gestation, and had complete data on maternal and neonatal outcomes. Women were excluded if they had pre-existing diabetes mellitus (type 1 or type 2), chronic hypertension, multiple pregnancies, fetal anomalies, delivery before 28 weeks of gestation or incomplete outcome data. A total of 15,245 women met the eligibility criteria and were included in the final analysis (**Figure 1**).

### Exposure definition and group classification

Early-pregnancy FBG was measured as part of routine prenatal care at the first antenatal visit (< 14 weeks of gestation) after an overnight fast of at least 8 hours. All FBG measurements were performed in a single central laboratory at Guangxi Zhuang Autonomous Region People’s Hospital using the hexokinase method on a Beckman Coulter AU5800 automated analyzer. The laboratory participated in the National External Quality Assessment Scheme (NEQAS) for Clinical Chemistry and maintained consistent quality control throughout the study period (2018–2022). GDM was diagnosed at 24–28 weeks of gestation using a 75-g OGTT according to the International Association of Diabetes and Pregnancy Study Groups (IADPSC) criteria, with GDM diagnosed if one or more of the following thresholds were met or exceeded: fasting glucose ≥ 5.1 mmol/L, 1-hour glucose ≥ 10.0 mmol/L, or 2-hour glucose ≥ 8.5 mmol/L (13).

Participants were categorized into four mutually exclusive groups based on early-pregnancy FBG and subsequent GDM status:

1. Normoglycemia group: FBG <5.1 mmol/L without GDM.
2. GDM-Regression group: FBG ≥5.1 mmol/L without GDM.
3. Late-onset GDM group: FBG <5.1 mmol/L with GDM.
4. GDM-Maintained group: FBG ≥5.1 mmol/L with GDM.

The terms GDM-Regression and GDM-Maintained are adopted from the nomenclature of the TOBOGM trial (8).

### Outcome ascertainment

#### Primary outcome

The primary outcome was HDP, a composite of gestational hypertension and preeclampsia. Gestational hypertension was defined as systolic blood pressure ≥140 mmHg and/or diastolic blood pressure ≥90 mmHg on at least two occasions at least 4 hours apart after 20 weeks of gestation in women with previously normal blood pressure. Preeclampsia was defined as gestational hypertension with proteinuria (≥300 mg/24 hours or protein/creatinine ratio ≥0.3) or, in the absence of proteinuria, new-onset thrombocytopenia, renal insufficiency, impaired liver function, pulmonary edema, or cerebral or visual symptoms, in accordance with the criteria of the International Society for the Study of Hypertension in Pregnancy (ISSHP) (14).

#### Secondary outcomes

Secondary outcomes included maternal outcomes (maternal anemia, defined as hemoglobin <110 g/L during pregnancy; cesarean section; abruptio placentae; placenta previa; premature rupture of membranes; and postpartum hemorrhage) and neonatal outcomes (birth weight; gestational age at delivery; infant sex; SGA and LGA, defined as birth weight <10th and >90th percentile for gestational age, respectively (15); preterm birth, defined as delivery at 28 to <37 completed weeks of gestation (16); macrosomia, defined as birth weight ≥4,000 g; low birth weight, defined as birth weight <2,500 g; umbilical artery pH; neonatal asphyxia, defined as Apgar score <7 at 5 minutes or umbilical artery pH <7.0; and stillbirth). Data were abstracted from electronic medical records by trained research staff blinded to exposure status.

### Covariates

Covariates were selected *a priori* based on clinical knowledge and review of the literature. Maternal characteristics included age (continuous, years), pre-pregnancy body mass index (BMI, continuous, calculated as weight in kilograms divided by height in meters squared), and age classification (<35 vs ≥35 years). BMI was categorized according to Chinese criteria: underweight (<18.5 kg/m²), normal (18.5–23.9 kg/m²), overweight (24.0–27.9 kg/m²), and obese (≥28.0 kg/m²) (17). Sociodemographic factors included marital status (married vs unmarried), employment status (employed vs unemployed), ethnicity (Han, Zhuang, or other minority), and educational level (junior high school or below, senior high school, undergraduate, graduate or above). Reproductive and medical history included parity (nulliparous vs multiparous), previous cesarean section (yes vs no), *in-vitro* fertilization (yes vs no), family history of diabetes mellitus (yes vs no), and smoking during pregnancy (yes vs no). Blood pressure measurements (systolic and diastolic) at the first antenatal visit were recorded for descriptive purposes but were not included as covariates in the multivariable models, as they were considered to lie on the causal pathway between GDM and HDP; adjusting for them could have introduced overadjustment bias.

### Statistical analysis

Normality of continuous variables was assessed by visual inspection of histograms and Q-Q plots. Continuous variables with skewed distributions were compared using the Kruskal–Wallis test, while those with approximately normal distributions were compared using one-way analysis of variance (ANOVA). Categorical variables were compared using the chi-square test, or Fisher’s exact test when expected cell counts were <5. Trends across ordered groups were assessed using linear regression for continuous variables and the Cochran–Armitage trend test for categorical variables. Pairwise comparisons were performed using Dunn’s test with Bonferroni correction for the Kruskal–Wallis test and Tukey’s honestly significant difference (HSD) test for ANOVA, as appropriate. For pairwise comparisons, a Bonferroni-adjusted *P* < 0.05 was considered statistically significant.

Multivariable logistic regression was used to estimate the association between the four-group classification and each outcome, with the Normoglycemia group as the reference. Crude and adjusted models were constructed, with the latter adjusting for a priori-selected confounders: maternal age (continuous), pre-pregnancy BMI (continuous), ethnicity, marital status, employment, parity, previous cesarean section, IVF, and family history of diabetes mellitus. Model fit was assessed using the Hosmer–Lemeshow goodness-of-fit test (with *P* > 0.05 indicating adequate calibration) and the Akaike Information Criterion (AIC). All adjusted models demonstrated adequate calibration (**Supplementary Table 1**), with consistently lower AIC values compared with crude models, indicating improved model fit after covariate adjustment.

The dose–response relationship between early-pregnancy FBG and HDP, as well as its subtypes (gestational hypertension and preeclampsia), was examined using restricted cubic splines with four knots placed at the 5th, 35th, 65th, and 95th percentiles of the FBG distribution, with knot locations selected based on minimizing AIC. For each model, overall P values and P for nonlinearity were calculated using ANOVA. When nonlinearity was statistically significant (*P* for nonlinearity <0.05), the inflection point was identified using the first derivative of the spline function. Odds ratios were referenced to FBG = 4.5 mmol/L, a value chosen *a priori* as it represents a clinically meaningful threshold near the center of the FBG distribution. Stratified analyses were performed by age (<35 vs ≥35 years), pre-pregnancy BMI (<24 vs ≥ 24 kg/m²), ethnicity (Han vs minority), and parity (nulliparous vs multiparous). Interaction P values were calculated using likelihood ratio tests comparing models with and without multiplicative interaction terms.

Sensitivity analyses were conducted using alternative FBG cutoffs (≥5.3 mmol/L and ≥5.6 mmol/L) to reassign GDM groups, and all multivariable regression analyses were repeated to assess the robustness of the findings.

All statistical tests were two-sided, and analyses were performed using R version 4.5.1 (R Foundation for Statistical Computing, Vienna, Austria).

## Result

### Study population and baseline characteristics

A total of 15,245 women with singleton pregnancies were included in the final analysis. Based on early-pregnancy FBG and subsequent GDM status, participants were categorized into four groups: Normoglycemia (FBG <5.1 mmol/L without GDM, *n* = 11,143, 73.1%), GDM-Regression (FBG ≥5.1 mmol/L without GDM, *n* = 1,201, 7.9%), Late-onset GDM (FBG <5.1 mmol/L with GDM, *n* = 2,308, 15.1%), and GDM-Maintained (FBG ≥5.1 mmol/L with GDM, *n* = 593, 3.9%).

Baseline characteristics across the four groups are presented in **Table 1**. Significant increasing trends across the four GDM groups were observed for maternal age, pre-pregnancy BMI, marital status, multiparity, previous cesarean section, IVF conception and family history of diabetes mellitus (all *P* for trend <0.001). In contrast, no significant trend was observed for ethnicity, educational level, or smoking status (*P* for trend >0.05 for all).

**Table 1:** Baseline Characteristics of Participants by early-pregnancy FBG and subsequent GDM status.

Variable	Normoglycemia (*n* = 11,143)	GDM-Regression (*n* = 1,201)	Late-onset GDM (*n* = 2308)	GDM-Maintained (*n* = 593)	*P*	*P* for trend
Age (years, $\bar{x}$±*s*)	31.09 ± 4.39^bcd^	32.08 ± 4.32^acd^	32.53 ± 4.32^abd^	33.69 ± 4.43^abc^	**<0.001**	**<0.001**
Age classification [*n* (%)]					**<0.001**	**<0.001**
<35 years	8779 (78.8) ^bcd^	838 (69.8) ^ad^	1552 (67.2) ^ad^	337 (56.8) ^abc^		
≥35 years	2364 (21.2)	363 (30.2)	756 (32.8)	256 (43.2)		
pre-BMI [kg/m^2^, *M* (*P_25_*, *P_75_*)]	21.23 (19.56−23.24) ^bcd^	22.06 (20.2−24.39) ^ad^	22.03 (20.28−24.22) ^ad^	23.50 (21.43−26.29) ^abc^	**<0.001**	**<0.001**
BMI category [*n* (%)]					**<0.001**	**<0.001**
Underweight	1327 (11.9) ^bcd^	88 (7.3) ^ad^	171 (7.4) ^ad^	20 (3.4) ^abc^		
Normal	7728 (69.4)	770 (64.1)	1512 (65.5)	304 (51.3)		
Overweight	1700 (15.3)	262 (21.8)	521 (22.6)	186 (31.4)		
Obese	388 (3.5)	81 (6.7)	104 (4.5)	83 (14)		
Marital status [*n* (%)]					**<0.001**	**<0.001**
Unmarried	1120 (10.1) ^cd^	100 (8.3)	186 (8.1) ^a^	39 (6.6) ^a^		
Married	10023 (89.9)	1101 (91.7)	2122 (91.9)	554 (93.4)		
Ethnicity [*n* (%)]					**0.008**	0.360
Han	6285 (56.4) ^b^	741 (61.7) ^ac^	1289 (55.8) ^b^	351 (59.2)		
Zhuang	4269 (38.3)	395 (32.9)	904 (39.2)	209 (35.2)		
Minority	589 (5.3)	65 (5.4)	115 (5)	33 (5.6)		
Employed [*n* (%)]					**<0.001**	**0.018**
No	2372 (21.3) ^bc^	303 (25.2) ^ac^	407 (17.6) ^ab^	125 (21.1)		
Yes	8771 (78.7)	898 (74.8)	1901 (82.4)	468 (78.9)		
Educational level [*n* (%)]					0.057	0.079
Junior high school	603 (5.4)	86 (7.2)	127 (5.5)	45 (7.6)		
Senior high school	1249 (11.2)	151 (12.6)	262 (11.4)	76 (12.8)		
Undergraduate	8263 (74.2)	848 (70.6)	1695 (73.4)	423 (71.3)		
Graduate and above	1028 (9.2)	116 (9.7)	224 (9.7)	49 (8.3)		
Family history of DM [*n* (%)]					**<0.001**	**<0.001**
No	10703 (96.1) ^cd^	1139 (94.8) ^cd^	2121 (91.9) ^abd^	519 (87.5) ^abc^		
Yes	440 (3.9)	62 (5.2)	187 (8.1)	74 (12.5)		
Parous [*n* (%)]					**<0.001**	**<0.001**
No	5957 (53.5) ^bd^	503 (41.9) ^a^	1225 (53.1) ^d^	250 (42.2) ^ac^		
Yes	5186 (46.5)	698 (58.1)	1083 (46.9)	343 (57.8)		
previous CS [*n* (%)]					**<0.001**	**<0.001**
No	9299 (83.5) ^bd^	945 (78.7) ^a^	1879 (81.4) ^d^	453 (76.4) ^ac^		
Yes	1844 (16.5)	256 (21.3)	429 (18.6)	140 (23.6)		
IVF [*n* (%)]					**<0.001**	**<0.001**
No	10484 (94.1) ^cd^	1138 (94.8) ^cd^	2122 (91.9) ^ab^	535 (90.2) ^ab^		
Yes	659 (5.9)	63 (5.2)	186 (8.1)	58 (9.8)		
Smoking [*n* (%)]					0.270^#^	0.536
No	11074 (99.4)	1189 (99.0)	2296 (99.5)	591 (99.7)		
Yes	69 (0.6)	12 (1.0)	12 (0.5)	2 (0.3)		
SBP (mmHg, $\bar{x}$±*s*)	106.90 ± 10.02^bcd^	108.49 ± 10.60^ac^	109.21 ± 10.38^abd^	112.30 ± 10.88 ^ac^	**<0.001**	**<0.001**
DBP (mmHg, $\bar{x}$±*s*)	66.06 ± 7.68^bcd^	68.06 ± 7.90 ^ac^	67.48 ± 7.91^abd^	70.17 ± 8.15^ac^	**<0.001**	**<0.001**
FBG (mmol/L, $\bar{x}$±*s*)	4.60 (4.39-4.78) ^bcd^	5.24 (5.16-5.39) ^ac^	4.64 (4.41-4.84) ^abd^	5.19 (5.19-5.48) ^ac^	**<0.001**	**<0.001**

Data are presented as mean (SD) or median (IQR) for continuous variables, and *n* (%) for categorical variables. Group comparisons were performed using the Kruskal-Wallis test for continuous variables. For categorical variables, the Chi-square test was used when expected frequencies were ≥ 5 in all cells; otherwise, ^#^Fisher’s exact test was applied. *P* for trend was calculated by linear regression or the Cochran-Armitage trend test. Superscript letters (a, b, c, d) indicate significant pairwise differences compared with the Normoglycemia, GDM-Regression, Late-onset GDM, and GDM-Maintained groups, respectively, after Bonferroni adjustment (adjusted *P* < 0.05).

GDM, gestational diabetes mellitus; SD, standard deviation; IQR, interquartile range; SBP, systolic blood pressure; DBP, diastolic blood pressure; BMI, body mass index; FBG, fasting blood glucose; IVF, *In-vitro* fertilization.

Bold values denote statistical significance (P < 0.05) as standard.

Systolic and diastolic blood pressures increased progressively across groups (both *P* for trend <0.001). As expected by the grouping definition, early-pregnancy FBG levels were significantly higher in the GDM-Regression and GDM-Maintained groups compared with the Normoglycemia and Late-onset GDM groups, with pairwise differences indicated in **Table 1**.

### Primary outcome: hypertensive disorders of pregnancy

The incidence of HDP increased progressively across the four groups: Normoglycemia 4.6%, GDM-Regression 6.9%, Late-onset GDM 8.1%, and GDM-Maintained 11.0% (*P* for trend < 0.001) (**Table 2**). Among HDP subtypes, the incidence of preeclampsia followed a similar pattern, with rates of 2.7%, 4.2%, 4.5%, and 6.7%, respectively, while gestational hypertension occurred in 1.9%, 2.7%, 3.6%, and 4.2%, respectively.

**Table 2:** Maternal and Neonatal Outcomes by early-pregnancy FBG and subsequent GDM status.

Outcomes	Normoglycemia (*n* = 11,143)	GDM-Regression (*n* = 1,201)	Late-onset GDM (*n* = 2308)	GDM-Maintained (*n* = 593)	*P*	*P* for trend
Primary Outcome						
HDP [*n* (%)]	517 (4.6) ^bcd^	83 (6.9) ^ad^	187 (8.1) ^a^	65 (11.0) ^ab^	**<0.001**	**<0.001**
GH	214 (1.9)	33 (2.7)	82 (3.6)	25 (4.2)		
PE	303 (2.7)	50 (4.2)	105 (4.5)	40 (6.7)		
Secondary outcomes						
Maternal outcome						
Maternal anemia [*n* (%)]	3912 (35.1) ^cd^	380 (31.6) ^cd^	1007 (43.6) ^ab^	264 (44.5) ^ab^	**<0.001**	**<0.001**
Abruptio placentae [*n* (%)]	244 (2.2)	25 (2.1)	47 (2.0)	17 (2.9)	0.666^#^	0.799
Placenta previa [*n* (%)]	146 (1.3)	15 (1.2)	33 (1.4)	10 (1.7)	0.845^#^	0.457
PROM [*n* (%)]	2401 (21.5)	242 (20.1)	518 (22.4)	132 (22.3)	0.452	0.461
PPH [*n* (%)]	510 (4.6)	58 (4.8)	101 (4.4)	24 (4.0)	0.864	0.560
Mode of delivery [*n* (%)]					**<0.001**	**<0.001**
Vaginal delivery	7305 (65.6) ^bcd^	720 (60.0) ^a^	1390 (60.2) ^a^	330 (55.6) ^a^		
Cesarean section	3838 (34.4)	481 (40.0)	918 (39.8)	263 (44.4)		
Neonatal outcome						
Birth weight [kg, *M* (*P*_25_, *P*_75_)]	3.19 (2.93−3.45) ^bd^	3.28 (3.00−3.53) ^ac^	3.20 (2.95−3.45) ^bd^	3.28 (2.99−3.56) ^ac^	**<0.001**	**<0.001**
Gestational age [w, *M* (*P*_25_, *P*_75_)]	39.29 (38.57−40.00) ^cd^	39.29 (38.43−40.00) ^cd^	39.14 (38.43−39.86) ^ab^	39.14 (38.14−39.86) ^ab^	**<0.001**	**<0.001**
Infant sex [*n* (%)]					**0.032**	0.076
Female	5273 (47.3)	527 (43.9)	1084 (47.0)	255 (43.0)		
Male	5870 (52.7)	674 (56.1)	1224 (53.0)	338 (57.0)		
SGA [*n* (%)]	1120 (10.1) ^b^	74 (6.2) ^ac^	210 (9.1) ^b^	43 (7.3)	**<0.001**	**0.002**
LGA [*n* (%)]	945 (8.5) ^bd^	169 (14.1) ^ac^	236 (10.2) ^bd^	110 (18.5) ^ac^	**<0.001**	**<0.001**
PTB [*n* (%)]	311 (2.8) ^d^	48 (4.0)	65 (2.8) ^d^	33 (5.6) ^ac^	**<0.001**	**0.008**
Macrosomia [*n* (%)]	274 (2.5) ^bd^	57 (4.7) ^a^	73 (3.2) d	38 (6.4) ^ac^	**<0.001**	**<0.001**
LBW [*n* (%)]	359 (3.2)	31 (2.6)	84 (3.6)	19 (3.2)	0.414	0.593
pH value [*M* (*P*_25_, *P*_75_)]	7.27 (7.22−7.31)	7.27 (7.23−7.331)	7.27 (7.22−7.31)	7.27 (7.23−7.32)	0.265	0.593
Neonatal asphyxia [*n* (%)]	108 (1.0)	10 (0.8)	30 (1.3)	13 (2.2)	**0.021^#^**	**0.010**
Stillbirth [*n* (%)]	8 (0.1)	1 (0.1)	3 (0.1)	1 (0.2)	0.860^#^	0.270

Data are presented as median (IQR) for continuous variables and n (%) for categorical variables. Group comparisons for categorical variables were performed using the Chi-square test, or ^#^Fisher’s exact test when expected cell counts were <5. P for trend was calculated using the Cochran-Armitage trend test. Superscript letters (a, b, c, d) indicate significant pairwise differences compared with the Normoglycemia, GDM-Regression, Late-onset GDM, and GDM-Maintained groups, respectively, after Bonferroni adjustment (adjusted *P* < 0.05). Statistical methods are detailed in the **Table 1** footnote.

GDM, gestational diabetes mellitus; HDP, hypertensive disorders of pregnancy; GH, gestational hypertension; PE, preeclampsia; PROM, premature rupture of membranes; PPH, postpartum hemorrhage; SGA, small for gestational age; LGA, large for gestational age; PTB, preterm birth; LBW, low birth weight.

Bold values denote statistical significance (P < 0.05) as standard.

Multivariable logistic regression analysis (**Table 3**) demonstrated that, compared with the Normoglycemia group, the adjusted odds ratios (*aOR*) for HDP were 1.32 (95% *CI*: 1.03–1.68; *P* = 0.027) for GDM-Regression, 1.53 (95% *CI*: 1.28–1.83; *P* < 0.001) for Late-onset GDM, and 1.65 (95% *CI*: 1.23–2.18; *P* < 0.001) for GDM-Maintained. GDM-Maintained was associated with the highest risk of HDP, with an approximately 1.65-fold increased risk compared with the Normoglycemia group.

**Table 3:** Association between GDM status and maternal and neonatal outcomes.

Outcomes	Groups	Crude model			Adjusted model†		
		*cOR*	*95% CI*	*P*	*aOR*	*95% CI*	*P*
Primary Outcome							
HDP	Normoglycemia	1.00	Reference		1.00	Reference	
	GDM-Regression	1.53	1.19-1.93	**<0.001**	1.32	1.03-1.68	**0.027**
	Late-onset GDM	1.81	1.52-2.15	**<0.001**	1.53	1.28-1.83	**<0.001**
	GDM-Maintained	2.53	1.91-3.30	**<0.001**	1.65	1.23-2.18	**<0.001**
Secondary outcomes							
Maternal outcomes							
Maternal anemia	Normoglycemia	1.00	Reference		1.00	Reference	
	GDM-Regression	0.86	0.752-0.97	**0.017**	0.89	0.78-1.02	0.085
	Late-onset GDM	1.43	1.31-1.57	**<0.001**	1.40	1.27-1.53	**<0.001**
	GDM-Maintained	1.48	1.26-1.75	**<0.001**	1.51	1.27-1.79	**<0.001**
Cesarean section	Normoglycemia	1.00	Reference		1.00	Reference	
	GDM-Regression	1.27	1.13-1.44	**<0.001**	1.15	0.98-1.34	0.081
	Late-onset GDM	1.26	1.15-1.38	**<0.001**	1.06	0.951.19	0.287
	GDM-Maintained	1.52	1.28-1.79	**<0.001**	1.04	0.84-1.29	0.697
Neonatal outcomes							
SGA	Normoglycemia	1.00	Reference		1.00	Reference	
	GDM-Regression	0.59	0.46-0.74	**<0.001**	0.69	0.53-0.87	**0.003**
	Late-onset GDM	0.90	0.77-1.04	0.163	0.94	0.80-1.10	0.423
	GDM-Maintained	0.70	0.50-0.95	**0.027**	0.91	0.65-1.25	0.583
LGA	Normoglycemia	1.00	Reference		1.00	Reference	
	GDM-Regression	1.77	1.48-2.10	**<0.001**	1.45	1.21-1.74	**<0.001**
	Late-onset GDM	1.23	1.06-1.43	**0.007**	1.10	0.94-1.28	0.229
	GDM-Maintained	2.46	1.97-3.04	**<0.001**	1.68	1.33-2.10	**<0.001**
Preterm birth	Normoglycemia	1.00	Reference		1.00	Reference	
	GDM-Regression	1.45	1.05-1.96	**0.019**	1.37	0.99-1.86	**0.047**
	Late-onset GDM	1.01	0.76-1.31	0.947	0.95	0.71-1.23	0.685
	GDM-Maintained	2.05	1.40-2.92	**<0.001**	1.78	1.20-2.56	**0.003**
Macrosomia	Normoglycemia	1.00	Reference		1.00	Reference	
	GDM-Regression	1.98	1.46-2.63	**<0.001**	1.62	1.19-2.17	**0.002**
	Late-onset GDM	1.30	0.99-1.67	0.053	1.17	0.89-1.52	0.258
	GDM-Maintained	2.72	1.89-3.81	**<0.001**	1.81	1.24-2.57	**0.001**
Neonatal asphyxia	Normoglycemia	1.00	Reference		1.00	Reference	
	GDM-Regression	0.86	0.42-1.56	0.644	0.91	0.44-1.66	0.771
	Late-onset GDM	1.35	0.88-1.99	0.153	1.23	0.80-1.84	0.326
	GDM-Maintained	2.29	1.22-3.94	**0.005**	2.15	1.12-3.80	**0.013**

Data are presented as OR (95% CI) with the Normoglycemia group as reference. Statistically significant associations (*P* < 0.05) are indicated in bold. P values were calculated using Wald tests. All adjusted models showed adequate calibration (Hosmer-Lemeshow *P* > 0.05; see **Supplementary Table 1** for detailed model fit statistics).

† Adjusted for age (continuous), pre-pregnancy BMI (continuous), marital status, employment, ethnicity, family history of diabetes mellitus, parity, previous cesarean section, and *in vitro* fertilization.

GDM, gestational diabetes mellitus; OR, odds ratio; CI, confidence interval; HDP, hypertensive disorders of pregnancy; SGA, small for gestational age; LGA, large for gestational age.

**Figure 2:**
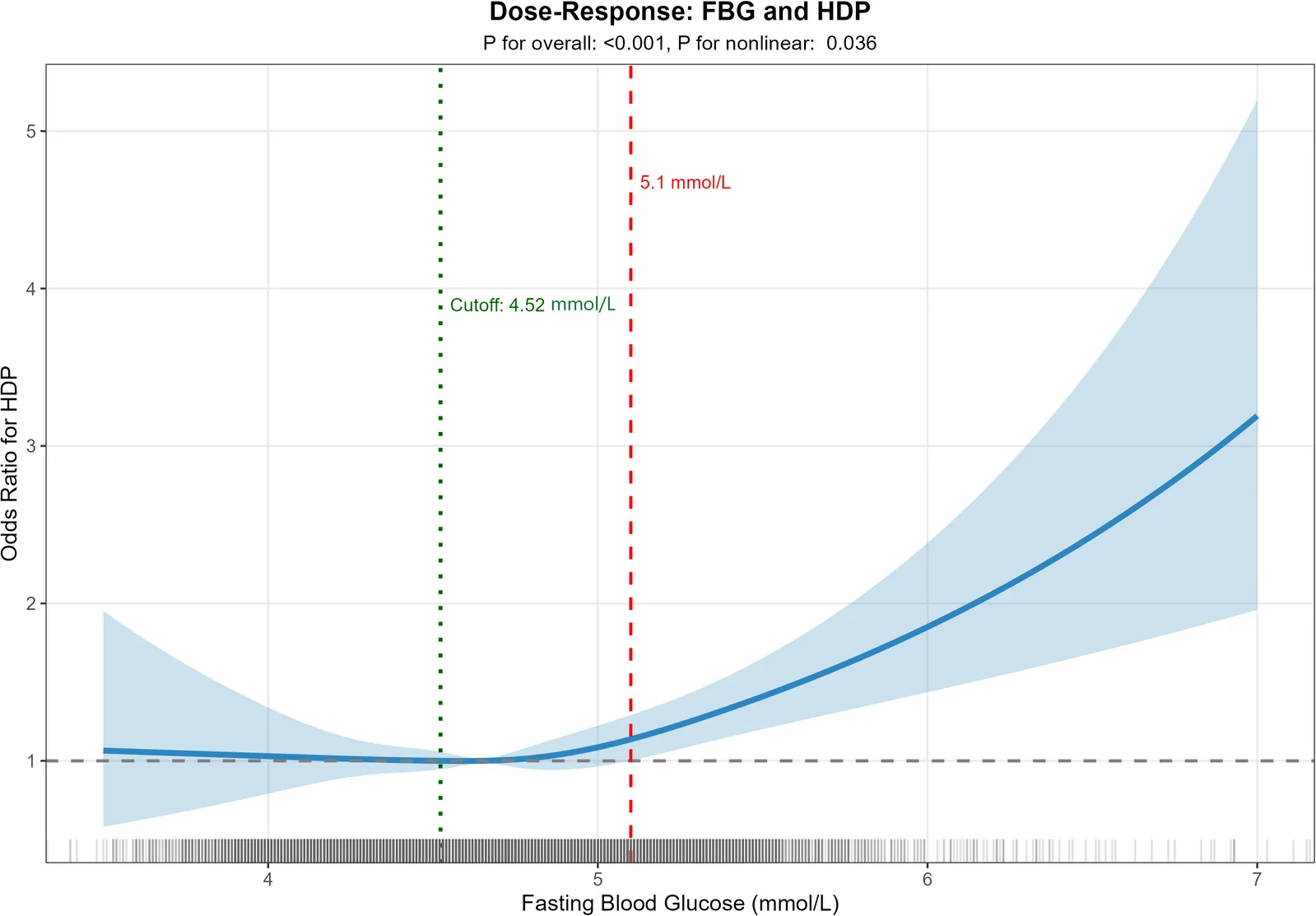
Dose-response relationship between early-pregnancy FBG and HDP. Restricted cubic spline regression with 4 knots (5th, 35th, 65th, and 95th percentiles) was used to model the association between early-pregnancy FBG and HDP. The model was adjusted for age, BMI, ethnicity, parity, previous CS, IVF, and family history of DM. Solid blue line: estimated OR; shaded area: 95% CI; gray dashed line: OR = 1 (reference); red dashed line: 5.1 mmol/L (clinical cutoff); green dotted line: 4.52 mmol/L (statistical inflection point).

**Figure 3:**
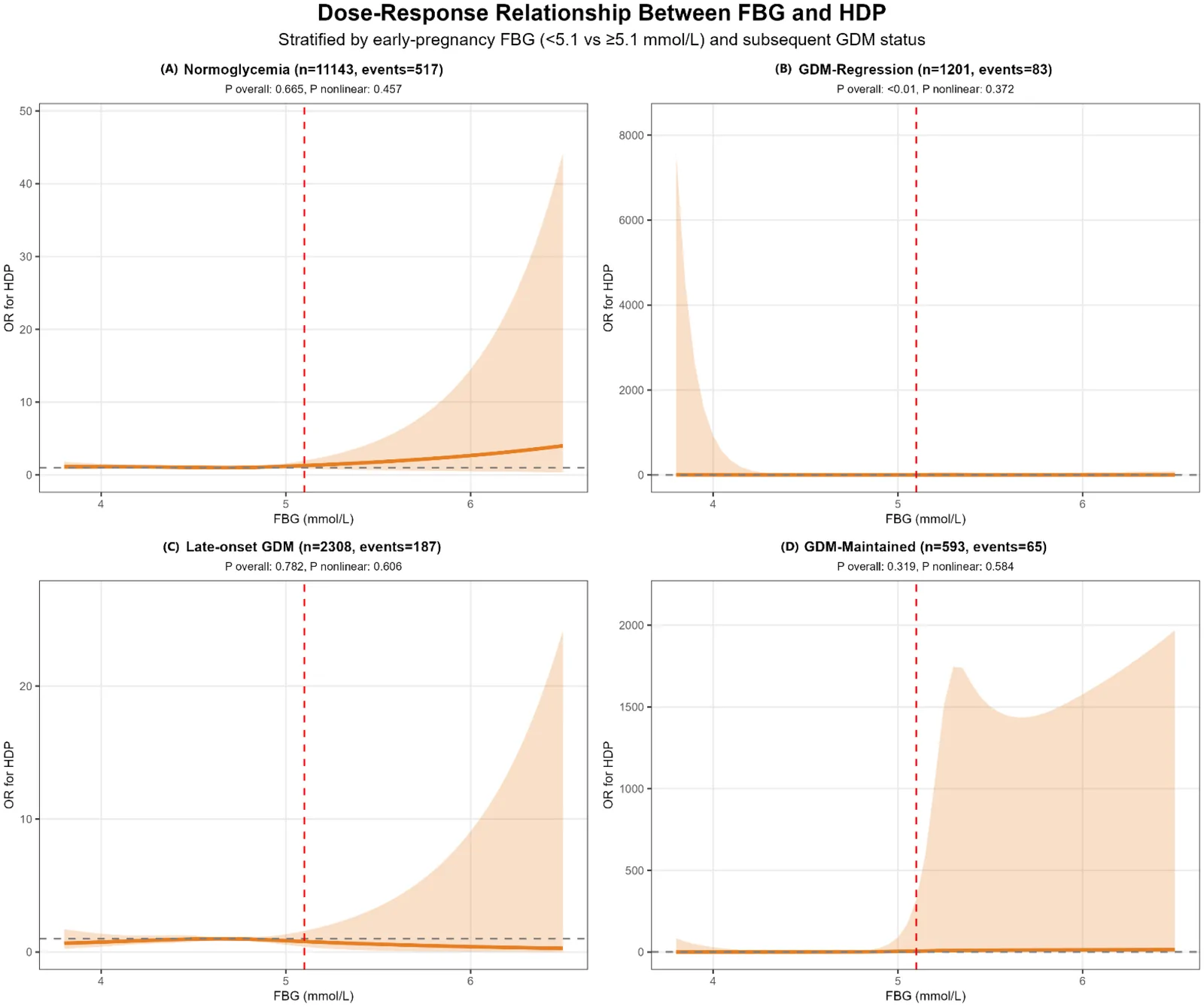
Dose-Response Relationship Between Early-Pregnancy FBG and HDP, Stratified by the Four-Group Classification. Restricted cubic spline (RCS) regression with 4 knots (5th, 35th, 65th, and 95th percentiles) was used to examine the association between early-pregnancy FBG and HDP stratified by the four-group classification: **(A)** Normoglycemia, **(B)** GDM-Regression, **(C)** Late-onset GDM, and **(D)** GDM-Maintained. The model was adjusted for age, BMI, ethnicity, parity, previous CS, IVF, and family history of DM. Solid orange line: estimated OR; shaded area: 95% CI; red dashed line: 5.1 mmol/L (clinical cutoff).

Restricted cubic spline analysis revealed a nonlinear dose-response relationship between early-pregnancy FBG and HDP (overall *P* < 0.001; *P* for nonlinearity = 0.036), with the risk increasing more steeply above approximately 5.1 mmol/L and a statistical inflection point at 4.52 mmol/L in our dataset (**Figure 2**). When stratified by four-group classification, the positive association between FBG and HDP was most apparent among women with GDM-Regression (overall *P* < 0.001), with no evidence of deviation from linearity (*P* for nonlinearity = 0.147). No significant associations were observed in the other three groups (all overall *P* > 0.05) (**Figure 3**). When examined by HDP subtypes, restricted cubic spline analysis showed a significant linear association between FBG and preeclampsia (overall *P* < 0.001; *P* for nonlinearity = 0.323), whereas no significant association was observed for gestational hypertension (overall *P* = 0.086; *P* for nonlinearity = 0.065) (**Supplementary Figure 1**).

### Secondary maternal outcomes

The incidence of maternal anemia varied significantly across the four groups, with the lowest rate observed in the GDM-Regression (31.6%), followed by the Normoglycemia (35.1%), Late-onset GDM (43.6%), and GDM-Maintained (44.5%) (*P* for trend <0.001) (**Table 2**). Compared with the Normoglycemia group, the risk of anemia was lower in the GDM-Regression group in the crude model (*cOR*: 0.86, 95% CI: 0.75–0.97; *P* = 0.017), although this association was attenuated and no longer statistically significant after full adjustment (*aOR*: 0.89, 95% CI: 0.78–1.02; *P* = 0.085). In contrast, Late-onset GDM (*aOR*: 1.40, 95% CI: 1.27–1.53; *P* < 0.001) and GDM-Maintained (*aOR*: 1.51, 95% CI: 1.27–1.79; *P* < 0.001) were both associated with a significantly increased risk of anemia (**Table 3**).

Cesarean section rates were higher in all GDM groups compared with the Normoglycemia group in crude analyses (all *P* < 0.001), with a progressive increase from the Normoglycemia group (34.4%) to the GDM-Regression (40.0%), Late-onset GDM (39.8%), and GDM-Maintained (44.4%) groups (*P* for trend <0.001) (**Table 2**). However, after full adjustment, these associations were substantially attenuated and no longer statistically significant, suggesting that the observed differences in crude analyses were largely explained by confounding factors.

No significant differences were observed across groups for other maternal outcomes, including abruptio placentae, placenta previa, premature rupture of membranes, or postpartum hemorrhage (all *P* > 0.05; **Table 2**).

### Secondary neonatal outcomes

Birth weight and gestational age at delivery differed significantly across the four groups. The highest median birth weights were observed in the GDM-Regression (3.28 kg) and GDM-Maintained (3.28 kg) groups, compared with the Normoglycemia (3.19 kg) and Late-onset GDM (3.20 kg) groups (*P* for trend <0.001). Gestational age at delivery was slightly shorter in the Late-onset and GDM-Maintained groups (median 39.14 weeks) compared with the Normoglycemia and GDM-Regression groups (both 39.29 weeks; *P* for trend <0.001). No significant difference was observed for infant sex across groups (*P* for trend = 0.076; **Table 2**).

The incidence of SGA was significantly lower in the GDM-Regression group (6.2%) than in the Normoglycemia group (10.1%). After full adjustment, the association remained significant (*aOR*: 0.69, 95% *CI*: 0.53–0.87; *P* = 0.003). No significant associations were observed for Late-onset GDM (*aOR*: 0.94, 95% *CI*: 0.80–1.10; *P* = 0.423) or GDM-Maintained (*aOR*: 0.91, 95% *CI*: 0.65–1.25; *P* = 0.583) (**Table 3**).

LGA and macrosomia showed consistent patterns across the four groups. Both outcomes had the highest incidence in the GDM-Maintained group (LGA: 18.5%; macrosomia: 6.4%), followed by the GDM-Regression group (LGA: 14.1%; macrosomia: 4.7%). Compared with the Normoglycemia group, the adjusted risks for LGA were significantly elevated in both the GDM-Regression (a*OR*: 1.45, 95% *CI*: 1.21–1.74; *P* < 0.001) and GDM-Maintained groups (*aOR*: 1.68, 95% *CI*: 1.33–2.10; *P* < 0.001), but not in the Late-onset GDM group (*aOR*: 1.10, 95% *CI*: 0.94–1.28; *P* = 0.229). Similarly, the adjusted risks for macrosomia were significantly elevated in the GDM-Regression *(aOR*: 1.62, 95% *CI*: 1.19–2.17; *P* = 0.002) and GDM-Maintained groups (*aOR*: 1.81, 95% *CI*: 1.24–2.57; *P* = 0.001), but not in the Late-onset GDM group (*aOR*: 1.17, 95% *CI*: 0.89–1.52; *P* = 0.258).

Preterm birth was most prevalent in the GDM-Maintained group (5.6%), followed by the GDM-Regression group (4.0%). After adjustment, GDM-Regression showed a borderline association with preterm birth (*aOR*: 1.37, 95% *CI*: 0.99–1.86; *P* = 0.047), while GDM-Maintained was associated with a significantly increased risk (*aOR*:1.78, 95% *CI*: 1.20–2.56; *P* = 0.003). Late-onset GDM was not associated with preterm birth (*aOR*: 0.95, 95% *CI*: 0.71–1.23; *P* = 0.685).

Neonatal asphyxia occurred infrequently across all groups (0.8%–2.2%). GDM-Maintained was associated with more than a two-fold increased risk of neonatal asphyxia (*aOR*: 2.15, 95% CI: 1.12–3.80; *P* = 0.013), whereas no significant associations were observed for GDM-Regression (*aOR*: 0.91, 95% CI: 0.44–1.66; *P* = 0.771) or Late-onset GDM (*aOR*: 1.23, 95% *CI*: 0.80–1.84; *P* = 0.326). Low birth weight and stillbirth did not differ significantly across groups (all *P* > 0.05; **Table 2**).

### Subgroup and stratified analyses

**Figure 4:**
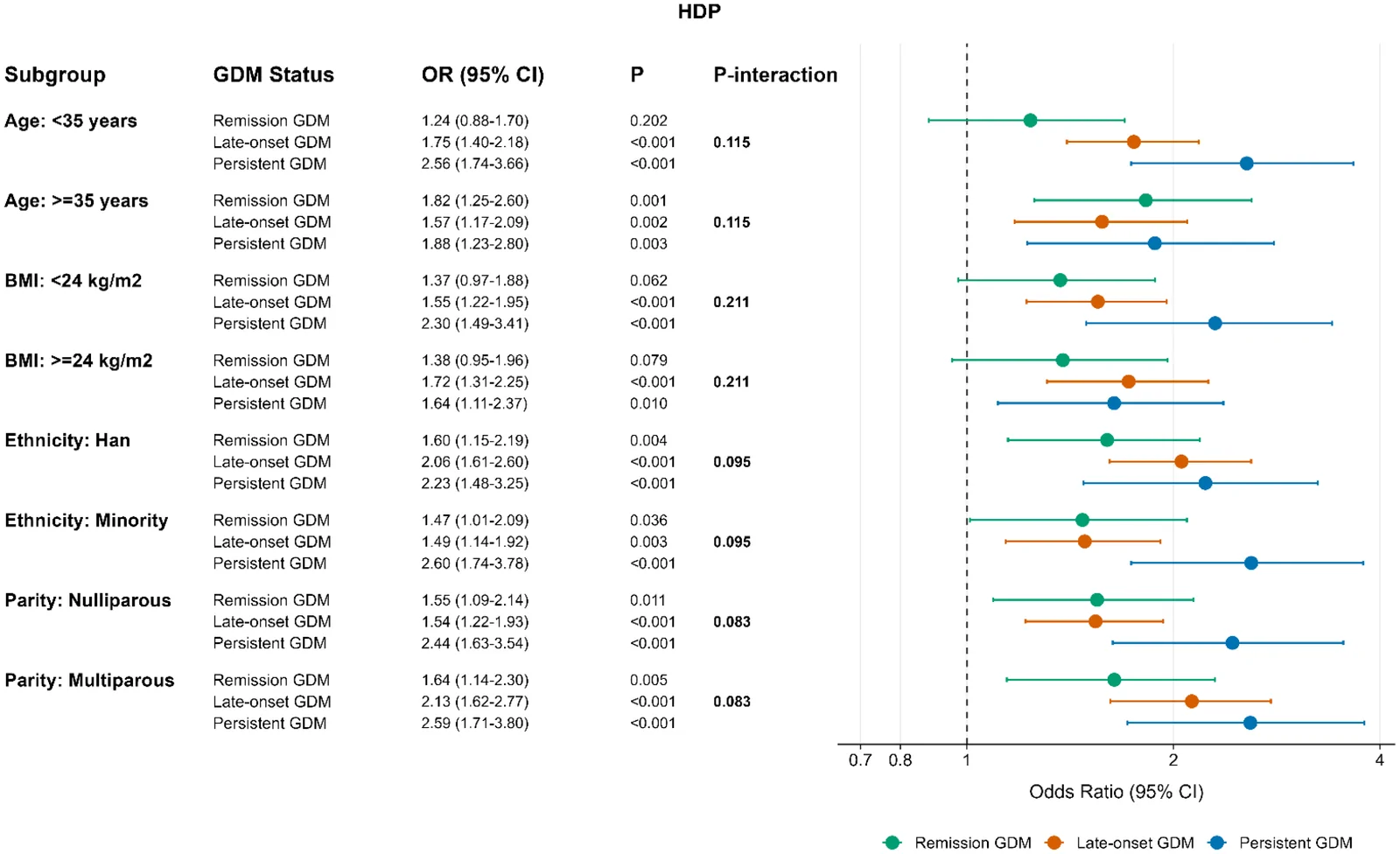
Stratified analysis of the association between GDM status and HDP by age, body mass index, ethnicity, and parity. odds ratios (ORs) and 95% confidence intervals (CIs) for the association between GDM status (GDM-Regression, Late-onset GDM, and GDM-Maintained) and HDP, stratified by age (<35 vs ≥35 years), BMI (<24 vs ≥24 kg/m²), ethnicity (Han vs Non-Han), and parity (nulliparous vs multiparous). The Normoglycemia group was the reference. Models were adjusted for age, BMI, ethnicity, parity, previous CS, IVF, and family history of DM, except the stratification variable. Dots represent ORs; horizontal lines indicate 95% CIs. P for interaction values are shown. GDM indicates gestational diabetes mellitus; HDP, hypertensive disorders of pregnancy; BMI, body mass index; CS, caesarean section; IVF, *in-vitro* fertilization; OR, odds ratio; CI, confidence interval.

Stratified analyses showed no evidence of significant effect modification by age (<35 vs ≥35 years), pre-pregnancy BMI (<24 vs ≥24 kg/m²), ethnicity (Han vs minority), and parity (nulliparous vs multiparous) (all *P* for interaction > 0.05), indicating that the association between FBG and HDP was generally consistent across these subgroups. Although point estimates for GDM-Maintained were somewhat higher among minority women (*aOR*: 2.60), multiparous women (*aOR*: 2.59), and women aged < 35 years (*aOR*: 2.56), the interaction tests were not statistically significant (**Figure 4**).

Stratified analyses for secondary maternal outcomes—cesarean delivery and maternal anemia—were also performed across age, BMI, ethnicity, and parity subgroups (**Supplementary Figures 2**, **3**). For cesarean section, a significant interaction was observed for BMI (*P* = 0.010), suggesting potential effect modification by pre-pregnancy BMI, whereas no significant interactions were found for age (*P* = 0.225), parity (*P* = 0.287), or ethnicity (*P* = 0.031). For maternal anemia, no significant effect modification was observed across any stratification variable (all *P* for interaction > 0.05), indicating that the associations between GDM status and anemia were consistent across these subgroups.

Stratified analyses for secondary neonatal outcomes—LGA, SGA, and preterm birth—were also performed across strata defined by age, BMI, ethnicity, and parity (**Supplementary Figures 4**–**6**). For LGA, the associations between GDM status and LGA were generally consistent across strata, although a nominally significant interaction was observed for age (*P* = 0.023) and BMI (*P* = 0.034), with no evidence of effect modification by parity (*P* = 0.491) or ethnicity (*P* = 0.427). For preterm birth, no significant interactions were observed across any stratification variable (all *P* for interaction > 0.05). Similarly, for SGA, the associations were consistent across all subgroups, with no significant interactions observed for age (*P* = 0.255), BMI (*P* = 0.177), parity (*P* = 0.337), or ethnicity (*P* = 0.237), indicating that the protective effect of GDM-Regression against SGA was robust across these clinically relevant subgroups.

### Sensitivity analyses

To assess the robustness of our findings, we repeated the multivariable analyses using alternative FBG cutoffs (≥5.3 mmol/L and ≥5.6 mmol/L) to define GDM-Regression and GDM-Maintained (**Table 4**).

**Table 4:** Sensitivity Analysis of the Association Between GDM Status and Maternal and Neonatal Outcomes Using Different FBG Cutoffs.

Outcomes	Groups	FBG≥5.3 mmol/L			FBG≥5.6 mmol/L		
		*aOR*	95% *CI*	*P*	*aOR*	95% *CI*	*P*
Primary outcome							
HDP	Normoglycemia	1.00	Reference		1.00	Reference	
	GDM-Regression	1.25	0.85-1.77	0.241	1.90	1.044-3.25	**0.026**
	Late-onset GDM	1.47	1.24-1.74	**<0.001**	1.49	1.26-1.75	**<0.001**
	GDM-Maintained	1.91	1.33-2.70	**<0.001**	2.31	1.32-3.87	**0.002**
Secondary outcomes							
*Maternal outcomes*							
Maternal anemia	Normoglycemia	1.00	Reference		1.00	Reference	
	GDM-Regression	0.85	0.69-1.03	0.098	0.73	0.49-1.07	0.117
	Late-onset GDM	1.42	1.30-1.55	**<0.001**	1.43	1.31-1.56	**<0.001**
	GDM-Maintained	1.50	1.19-1.90	**0.001**	1.48	0.99-2.20	0.056
Cesarean section	Normoglycemia	1.00	Reference		1.00	Reference	
	GDM-Regression	1.28	1.02-1.62	**0.035**	2.53	1.64-3.89	**<0.001**
	Late-onset GDM	1.06	0.95-1.18	0.293	1.05	0.95-1.17	0.348
	GDM-Maintained	1.02	0.76-1.37	0.880	1.22	0.75-1.97	0.425
Neonatal outcomes							
Small for gestational age	Normoglycemia	1.00	Reference		1.00	Reference	
	GDM-Regression	0.63	0.42-0.92	**0.021**	0.65	0.27-1.31	0.274
	Late-onset GDM	0.93	0.80-1.09	0.376	0.96	0.82-1.11	0.564
	GDM-Maintained	1.12	0.72-1.68	0.599	1.03	0.45-2.02	0.941
Large for gestational age	Normoglycemia	1.00	Reference		1.00	Reference	
	GDM-Regression	1.29	0.97-1.684	0.073	1.99	1.26-3.06	**0.002**
	Late-onset GDM	1.14	0.98-1.31	0.079	1.15	1.00-1.32	**0.046**
	GDM-Maintained	1.52	1.11-2.06	**0.007**	2.05	1.24-3.28	**0.003**
Preterm birth	Normoglycemia	1.00	Reference		1.00	Reference	
	GDM-Regression	1.42	0.88-2.19	0.127	1.47	0.57-3.10	0.361
	Late-onset GDM	1.05	0.81-1.33	0.719	1.07	0.84-1.34	0.600
	GDM-Maintained	1.47	0.82-2.44	0.161	1.42	0.495-3.201)	0.453
Macrosomia	Normoglycemia	1.00	Reference		1.00	Reference	
	GDM-Regression	2.04	1.35-2.98	**<0.001**	2.29	1.10-4.25	**0.015**
	Late-onset GDM	1.20	0.93-1.53	0.152	1.20	0.95-1.51	0.126
	GDM-Maintained	1.93	1.19-2.98	**0.005**	2.47	1.17-4.66	**0.010**
Neonatal asphyxia	Normoglycemia	1.00	Reference		1.00	Reference	
	GDM-Regression	0.85	0.26-2.05	0.755	—	—	—
	Late-onset GDM	1.46	1.00-2.10	**0.044**	1.42	0.98-2.03	0.058
	GDM-Maintained	0.91	0.22-2.47	0.869	0.81	0.05-3.75	0.831

Data are presented as adjusted odds ratios (aOR) with 95% confidence intervals (CI) and P values. Models were adjusted for age (continuous), pre-pregnancy BMI (continuous), marital status, employment, ethnicity, family history of DM, parity, previous cesarean section, and *in vitro* fertilization. The Normoglycemia group served as the reference for all comparisons. FBG ≥5.3 mmol/L and FBG ≥5.6 mmol/L represent different diagnostic cutoffs for early-onset GDM. Statistically significant associations (*P* < 0.05) are indicated in bold. — indicates that the estimate was not estimable due to insufficient events in the subgroup. Model calibration was adequate in all sensitivity analyses (Hosmer-Lemeshow *P* > 0.05; data not shown).

GDM, gestational diabetes mellitus; FBG, fasting blood glucose; HDP, hypertensive disorders of pregnancy; aOR, adjusted odds ratio; CI, confidence interval.

The overall pattern of associations remained consistent across both cutoffs. Using the ≥5.3 mmol/L cutoff, the adjusted odds ratios for HDP were 1.25 (95% *CI*: 0.85–1.77; *P* = 0.241) for GDM-Regression, 1.47 (95% *CI*: 1.24–1.74; *P* < 0.001) for Late-onset GDM, and 1.91 (95% *CI*: 1.33–2.70; *P* < 0.001) for GDM-Maintained. Using the ≥5.6 mmol/L cutoff, the corresponding aORs were 1.90 (95% *CI*: 1.04–3.25; *P* = 0.026) for GDM-Regression, 1.49 (95% *CI*: 1.26–1.75; *P* < 0.001) for Late-onset GDM, and 2.31 (95% *CI*: 1.32–3.87; *P* = 0.002) for GDM-Maintained. GDM-Maintained remained the group with the highest risk of HDP across both sensitivity analyses. Notably, GDM-Regression showed a statistically significant association with HDP at the ≥5.6 mmol/L cutoff, whereas the association was not significant at the ≥5.3 mmol/L cutoff, suggesting a dose-response gradient with higher effect estimates at higher FBG thresholds.

For other outcomes, the sensitivity analyses confirmed the robustness of the main findings for the protective effect of GDM-Regression against SGA and the elevated risks of LGA and macrosomia associated with GDM-Maintained across both cutoffs. The association between GDM-Maintained and neonatal asphyxia was not significant in either sensitivity analysis.

## Discussion

In this large retrospective cohort study of 15,245 Chinese women, we evaluated the association between early-pregnancy fasting blood glucose and GDM status with maternal and neonatal outcomes using a four-group classification: Normoglycemia, GDM-Regression, Late-onset GDM, and GDM-Maintained. Our findings demonstrate a graded increase in HDP risk across the four groups, with GDM-Maintained conferring the highest risk (*aOR*: 1.65), followed by Late-onset GDM (*aOR*: 1.53) and GDM-Regression (*aOR*: 1.32). Restricted cubic spline analysis further revealed a nonlinear dose–response relationship between FBG and HDP, with a statistical inflection point at 4.52 mmol/L—a threshold below the current clinical cutoff of 5.1 mmol/L. These findings support the concept that pregestational dysglycemia represents an early marker of metabolic risk with implications extending beyond GDM diagnosis alone.

The graded association between the four-group classification and HDP aligns with emerging evidence linking preconception prediabetes to HDP. McCarthy et al., in a cohort of 14,302 adolescents and young adults, demonstrated that preconception HbA1c in the prediabetes range (5.7%–6.4%) was associated with an 18% increased risk of HDP (18). Our findings extend this observation by showing that the risk varies by GDM subtype and that the association between early-pregnancy FBG and HDP is nonlinear, with a potential inflection point at 4.52 mmol/L. Notably, the positive association between early-pregnancy FBG and HDP was most apparent in the GDM-Regression group, underscoring that women with early hyperglycemia who do not progress to GDM remain at elevated risk (10, 19).

When examined by subtypes, the dose-response relationship between early-pregnancy FBG and HDP differed: a significant linear association was observed for preeclampsia, whereas no significant association was found for gestational hypertension. This distinction is biologically plausible, as preeclampsia and gestational hypertension are thought to have partially distinct pathophysiological pathways, with endothelial dysfunction and placental ischemia playing more central roles in preeclampsia (20–22). Our findings are consistent with a recent large Chinese cohort study that reported an association between early-pregnancy glucose levels and preeclampsia risk (23). However, that study did not examine gestational hypertension separately. Our analysis addresses this gap by demonstrating that the association with early-pregnancy FBG was significant for preeclampsia but not for gestational hypertension.

Beyond the dose-response relationship between FBG and HDP subtypes, the categorical classification of glycemic trajectories may further refine risk stratification. The four-group classification revealed a more nuanced picture than previously appreciated, with distinct risk profiles for each of the four GDM groups. GDM-Maintained emerged as the highest-risk group across nearly all outcomes—HDP, LGA, macrosomia, preterm birth, and neonatal asphyxia—consistent with the concept that the combination of fasting and post-load hyperglycemia reflects the most adverse metabolic phenotype (24). GDM-Regression presented a more complex pattern: reduced SGA risk (*aOR*: 0.69) alongside elevated LGA (*aOR*: 1.45) and macrosomia (*aOR*: 1.62) risks. This dual pattern may reflect early metabolic programming, whereby transient early hyperglycemia promotes fetal growth without the deleterious effects of sustained hyperglycemia, and aligns with emerging evidence of metabolic heterogeneity across GDM subtypes (24). Notably, while the TOBOGM study showed that women with early GDM who regressed to normoglycemia had pregnancy outcomes similar to those without GDM (25), our GDM-Regression group—defined by elevated early-pregnancy FBG rather than OGTT-based early GDM and for whom information regarding early lifestyle or glucose-lowering interventions was unavailable—exhibited persistent risks of fetal overgrowth, suggesting that the natural history of glycemic trajectories may be modified by both definition and intervention timing (26). Late-onset GDM was primarily associated with HDP and maternal anemia, with less pronounced effects on neonatal outcomes, suggesting that the timing of hyperglycemia onset is a critical determinant of the risk profile (27, 28). These findings underscore the importance of distinguishing GDM subtypes and timing of onset in both clinical risk assessment and research.

The GDM-Regression and GDM-Maintained groups are distinguished by the subsequent OGTT results at 24–28 weeks, which capture post-load glycemic responses not reflected by early-pregnancy FBG alone. Women in the GDM-Regression group may represent a phenotype of transient early hyperglycemia that resolves as pregnancy advances, potentially reflecting early but adaptive insulin resistance. Alternatively, some degree of misclassification due to biological variability or measurement precision cannot be excluded. Nevertheless, the graded risk patterns observed across multiple outcomes, together with the consistency of findings in sensitivity analyses using alternative FBG cutoffs, support the notion that these groups may represent clinically distinguishable glycemic patterns, rather than being purely statistical artifacts.

Several biological mechanisms may explain these associations. The link between early hyperglycemia and HDP may be mediated through endothelial dysfunction, a hallmark of both prediabetes and preeclampsia. Experimental models of pregestational prediabetes have demonstrated elevated inflammatory markers, reduced placental growth factor and vascular endothelial growth factor receptor 1, and impaired nitric oxide bioavailability—all implicated in preeclampsia pathogenesis (29). Recent human data further support this pathway, showing that serum PLGF levels are already decreased in women with GDM prior to the diagnosis of glucose intolerance, suggesting that placental endothelial dysfunction may precede and contribute to both metabolic and hypertensive complications (30). The association between GDM and fetal overgrowth is well explained by the Pedersen hypothesis, whereby maternal hyperglycemia leads to fetal hyperinsulinemia and accelerated growth. This mechanism has been validated in large-scale contemporary cohorts, most notably the HAPO study, which demonstrated a continuous linear relationship between maternal glucose levels and both fetal insulinemia and neonatal adiposity across the full range of maternal glycemia (31). The finding that GDM-Maintained conferred the highest LGA and macrosomia risk is consistent with this mechanism. The reduced SGA risk in GDM-Regression may reflect a “reset” of the metabolic environment following early hyperglycemia, allowing for adequate nutrient delivery without the catabolic effects of sustained hyperglycemia—a hypothesis warranting further investigation. This speculative interpretation is supported by the observation that placental adaptations to maternal metabolic status can profoundly influence fetal growth trajectories, and that the timing and duration of metabolic stress are critical determinants of placental function and fetal outcomes (32).

Our study has several strengths. The large sample size (n=15,245) provided sufficient statistical power to detect associations across multiple outcomes. The four-group classification allowed for nuanced risk stratification, and the comprehensive outcome ascertainment covering both maternal and neonatal endpoints enabled a holistic assessment of pregnancy complications. The use of restricted cubic spline analysis provided insight into the dose–response relationship, and sensitivity analyses using alternative early-pregnancy FBG cutoffs confirmed the robustness of the main findings.

Several limitations should be acknowledged. First, this was a retrospective cohort study from a single center in China, which may limit generalizability to other populations. Second, the relatively small sample size of the GDM-Maintained group (n=593) may have limited statistical power for some subgroup analyses. Third, early-pregnancy FBG was measured at varying gestational ages (<14 weeks) and relied on a single measurement, which may be subject to biological variability and may not fully capture an individual’s baseline glycemic status. This could have led to some degree of non-differential misclassification, which would likely bias our effect estimates toward the null. Fourth, as a retrospective study, we did not have access to data on early-pregnancy interventions—such as dietary counseling, lifestyle modifications, glucose monitoring, or endocrinology referrals—that may have been offered to women with elevated early FBG, as there was no standardized protocol for early intervention during the study period. Such unmeasured interventions may have influenced subsequent OGTT results and pregnancy outcomes, and this should be considered when interpreting the findings, particularly for the GDM-Regression group. Fifth, although we adjusted for multiple confounders, residual confounding from unmeasured variables cannot be excluded, including lifestyle factors (dietary patterns and physical activity), gestational weight gain, metabolic parameters (HbA1c, insulin resistance indices, and lipid profiles), and clinical history (history of pre-eclampsia, family history of hypertension, and medication use including aspirin for pre-eclampsia prevention). Finally, as with all observational studies, causality cannot be established; moreover, this study evaluated associations rather than developing or validating an individual-level risk prediction model. Accordingly, our findings should not be interpreted as evidence of predictive performance or direct clinical utility.

Despite these limitations, the graded associations observed across the four glycemic groups offer several insights that may inform clinical risk assessment and future research. The progressive increase in HDP risk—from 4.6% in the Normoglycemia group to 11.0% in the GDM-Maintained group—suggests that early-pregnancy FBG measurement could be a useful component of prenatal risk assessment. Women with elevated early FBG, even those who do not subsequently develop GDM, may warrant enhanced surveillance for HDP and fetal overgrowth. The distinct risk profiles of GDM-Regression and-Maintained groups raise the hypothesis that these groups may benefit from differentiated approaches: GDM-Maintained may require more intensive glycemic management, whereas GDM-Regression might benefit from targeted monitoring for HDP.

The identified inflection point at 4.52 mmol/L, while statistically significant in our cohort, should be interpreted with caution. This finding has not been validated in independent populations. We emphasize that this value should not be used as a clinical cutoff for intervention at this stage. Rather, it serves as a hypothesis-generating observation that warrants further investigation and external validation.

Future research should focus on validating the four-group classification in prospective multicenter cohorts and determining whether early intervention in women with elevated early FBG can reduce adverse outcomes. In addition, whether the observed inflection point at 4.52 mmol/L could inform risk stratification warrants exploration in interventional studies.

In conclusion, this cohort study demonstrates that the four-group classification based on early-pregnancy FBG and subsequent GDM status may facilitate risk stratification for adverse pregnancy. GDM-Maintained is associated with the highest risk across most outcomes, while GDM-Regression carries modest but significant risks of HDP and fetal overgrowth, alongside protection against SGA. The observed inflection point at 4.52 mmol/L in the FBG-HDP relationship warrants further investigation and external validation. These results support the potential value of early-pregnancy glycemic assessment in refining risk stratification and guiding tailored surveillance strategies.

## Data Availability

The original contributions presented in the study are included in the article/**Supplementary Material**. Further inquiries can be directed to the corresponding author.
