# Supplementary material for: Early-pregnancy fasting blood glucose and subsequent GDM define four distinct risk groups for adverse pregnancy outcomes: a cohort study of 15,245 women
Source: Front Endocrinol (Lausanne). 2026 Aug 7;17:1936170. doi: 10.3389/fendo.2026.1936170 (PMC13493287; doi:10.3389/fendo.2026.1936170)
Supplement: Supplementary file 1 [file Table1.docx]

Supplementary materials

**Title:** Early-Pregnancy Fasting Blood Glucose and Subsequent GDM Define Four Distinct Risk Groups for Adverse Pregnancy Outcomes: A Cohort Study of 15,245 Women

**Supplementary Table S1. Model Fit Statistics for Logistic Regression Models**

| **Outcomes** | **Crude Model** | | | **Adjusted Model** | | |
| --- | --- | --- | --- | --- | --- | --- |
|  | **AIC** | **HL *χ²*** | **HL *P*** | **AIC** | **HL *χ²*** | **HL *P*** |
| Primary outcome |  |  |  |  |  |  |
| HDP | 6504.5 | NA^†^ | NA^†^ | 6210.1 | 5.285 | 0.152 |
| Maternal outcomes | |  |  |  |  |  |
| Anemia | 19927.8 | NA^†^ | NA^†^ | 19739.6 | 6.968 | 0.073 |
| Cesarean section | 19826.6 | NA^†^ | NA^†^ | 14443.7 | 2.435 | 0.487 |
| Neonatal outcomes | |  |  |  |  |  |
| SGA | 9549.1 | NA^†^ | NA^†^ | 9236.9 | 6.470 | 0.091 |
| LGA | 9546.8 | NA^†^ | NA^†^ | 9081.1 | 6.562 | 0.087 |
| Preterm birth | 4097.4 | NA^†^ | NA^†^ | 4095.6 | 2.691 | 0.442 |
| Macrosomia | 3968.7 | NA^†^ | NA^†^ | 3812.7 | 5.830 | 0.120 |
| Neonatal asphyxia | 1785.3 | NA^†^ | NA^†^ | 1736.8 | 2.756 | 0.431 |

Note: AIC, Akaike information criterion; HL, Hosmer-Lemeshow. All adjusted models showed adequate calibration (HL *P* > 0.05).

^†^ Hosmer-Lemeshow test could not be computed for crude models due to insufficient events per covariate.

**Supplementary Figure 1. Dose-Response Relationship Between Early-Pregnancy Fasting Blood Glucose and HDP Subtypes**

Note: Restricted cubic spline regression with four knots (5th, 35th, 65th, and 95th percentiles) was used to model the association between early-pregnancy FBG and HDP subtypes: A. gestational hypertension and B. preeclampsia. The model was adjusted for age, pre-pregnancy BMI, ethnicity, parity, previous cesarean section, IVF, and family history of DM. Solid blue lines represent estimated ORs; shaded areas indicate 95% CIs; the gray dashed line indicates the null value (OR=1); the red dashed line indicates the clinical cutoff of 5.1 mmol/L. For gestational hypertension, the overall association was not statistically significant (overall *P* = 0.086; *P* for nonlinearity = 0.065). For preeclampsia, the overall association was significant (overall *P* < 0.001), with no evidence of nonlinearity (*P* for nonlinearity = 0.323), indicating a linear dose–response relationship. The green dotted line (statistical inflection point) was not drawn because nonlinearity was not statistically significant in either analysis.

**Supplementary Figure 2. Stratified Analysis of the Association Between GDM Status and Maternal Anemia**

Note: Odds ratios (ORs) and 95% confidence intervals (CIs) for the association between GDM status (GDM-Regression, Late-onset GDM, and GDM-Maintained) and maternal anemia, stratified by age (<35 vs ≥35 years), BMI (<24 vs ≥24 kg/m²), ethnicity (Han vs non-Han), and parity (nulliparous vs multiparous). The Normoglycemia group served as the reference. Models were adjusted for age, pre-pregnancy BMI, ethnicity, parity, previous cesarean section, IVF, and family history of diabetes mellitus, except the stratification variable. Dots represent ORs; horizontal lines indicate 95% CIs. P for interaction values are shown for each stratification variable. GDM indicates gestational diabetes mellitus; BMI, body mass index; CS, cesarean section; IVF, in-vitro fertilization; OR, odds ratio; CI, confidence interval.

**Supplementary Figure 3. Stratified Analysis of the Association Between GDM Status and Caesarean section**

Note: Stratified analysis by age, BMI, ethnicity, and parity. The Normoglycemia group served as the reference. Dots represent ORs; horizontal lines indicate 95% CIs. P for interaction values are shown. For adjustment variables and subgroup definitions, see Supplementary Figure 2 legend.

**Supplementary Figure 4. Stratified Analysis of the Association Between GDM Status and Large for Gestational Age**

Note: Stratified analysis by age, BMI, ethnicity, and parity. The Normoglycemia group served as the reference. Dots represent ORs; horizontal lines indicate 95% CIs. P for interaction values are shown. For adjustment variables and subgroup definitions, see Supplementary Figure 2 legend.

**Supplementary Figure 5. Stratified Analysis of the Association Between GDM Status and Small for Gestational Age**

Note: Stratified analysis by age, BMI, ethnicity, and parity. The Normoglycemia group served as the reference. Dots represent ORs; horizontal lines indicate 95% CIs. P for interaction values are shown. For adjustment variables and subgroup definitions, see Supplementary Figure 2 legend.

**Supplementary Figure 6. Stratified Analysis of the Association Between GDM Status and Preterm Birth**

Note: Stratified analysis by age, BMI, ethnicity, and parity. The Normoglycemia group served as the reference. Dots represent ORs; horizontal lines indicate 95% CIs. P for interaction values are shown. For adjustment variables and subgroup definitions, see Supplementary Figure 2 legend.
